# Systems Pharmacology-Based Research on the Mechanism of Tusizi-Sangjisheng Herb Pair in the Treatment of Threatened Abortion

**DOI:** 10.1155/2020/4748264

**Published:** 2020-07-20

**Authors:** Ming Yang, Jianghe Luo, Yan Li, Limian Xu

**Affiliations:** ^1^School of Nursing, Guangzhou University of Chinese Medicine, Guangzhou, 510006 Guangzhou, China; ^2^School of Chemical Engineering, Dalian University of Technology, Dalian, 116024 Liaoning, China; ^3^Department of Gynecology and Obstetrics, Affiliated Hospital 1, Guangzhou University of Chinese Medicine, Guangzhou 510006, China

## Abstract

Threatened abortion (TA) is a common complication with high incidence in the first trimester of pregnancy, which will end in miscarriage if not treated properly. The Chinese herbs Cuscutae Semen (Tusizi in Chinese) and Herba Taxilli (Sangjisheng in Chinese) first recorded in the ancient classic medical book *Shennong Bencao Jing* are effective and widely used as an herb pair for the treatment of TA, while the active ingredients and the functional mechanism of Tusizi-Sangjisheng herb pair treating TA are still unknown. In order to exploit the relationship between those two herbs and TA, systems pharmacology analysis was carried out in this study. A total of 75 ingredients of Tusizi-Sangjisheng were collected from Traditional Chinese Medicine System Pharmacology Database and Analysis Platform (TCMSP). 12 bioactive compounds were screened, and 153 directly related targets were predicted by systematic models. Besides, Gene Ontology (GO) enrichment analysis and Kyoto Encyclopedia of Genes and Genomes (KEGG) pathway enrichment analysis were used to systematically explore the potential mechanisms of Tusizi-Sangjisheng treating TA. Meanwhile, Compound-Target (C-T), Target-Disease (T-D), and Target-Pathway (T-P) networks were constructed to further quest the underlying functional mechanisms of Tusizi-Sangjisheng. As a result, 31 targets and 3 key pathways were found to be directly related to TA that includes mitogen-activated protein kinases (MAPKs), phosphatidylinositol-3-kinase/protein kinase B (PI3K-Akt), and transforming growth factor-*β* (TGF-*β*) signaling pathways. The results in this study may provide some valuable clues about the molecular mechanisms of the efficient Chinese herb pair Tusizi-Sangjisheng in the treatment of TA.

## 1. Introduction

Threatened abortion (TA), defined as vaginal bleeding with or without lower abdominal pain or backache with a closed cervix and an intrauterine viable fetus, is the commonest complication that occurs in early pregnancy [1], especially in 8-12 gestational weeks when the secretion of estrogen and progesterone shifts from corpus luteum to placental [2] (during the shift period, a pregnant woman is prone to go through limited corpus luteum function or an abnormality of placental progesterone production and secretion). TA affects one in five pregnancies, and about half of the cases unfortunately end in miscarriage [3, 4]. Besides, studies have shown that experiencing TA in the first trimester is associated with high risk of experiencing adverse pregnancy outcomes, such as premature rupture of membranes, placenta previa, and low birth weight [5]. TA is the cumulative result of several complicated pathogenic factors. Chromosomal abnormality is the important cause of TA that accounts for about 50-70% of cases [6, 7]. In addition, uterine malformations, cervical incompetence, polycystic ovaries, poorly controlled diabetes mellitus, maternal infections, immune dysfunctions such as antiphospholipid syndrome, and exposure to environmental toxins also have association with TA [7–9].

In clinical practice, targeted and directed treatment should be used to prevent miscarriage when specific causes are identified. For example, combined aspirin and heparin are effective in TA induced by antiphospholipid syndrome while antibiotics are useful for TA induced by bacterial vaginosis [7] and surgical interventions such as cervical cerclage are alternative and efficient for cervical incompetence. However, about half of the women suffering from miscarriage have no certain causes to be identified [10], making the treatment empirical. Generally, bed rest, avoidance of sexual intercourse, and drugs for some specific manifestations are usually prescribed for a woman with symptoms of TA [2, 11]. For instance, increased uterine activity is considered to be related to TA and so tocolytic drugs like magnesium sulfate and phloroglucinol (PHL) are commonly used for treatment [12]. Besides, since low serum human chorionic gonadotropin (hCG) and progesterone level increases the risk of miscarriage, drugs like exogenous progesterone [13, 14] and estrogen [15] are usually administrated to treat TA. What is more, anti-D immunoglobulin [16] is recommended for RhD-negative women with TA. In China, TA can be mainly attributed to the deficiency of the viscera especially the deficiency of the kidney according to the theories of traditional Chinese medicine (TCM). Herbs like Cuscutae Semen (Tusizi in Chinese), Herba Taxilli (Sangjisheng in Chinese), Dipsaci Radix (Xuduan in Chinese), and Astragali Radix (Huangqi in Chinese) are used to treat TA for their functions of replenishing the kidney essence (*Shen Jing* in Chinese) and nourishing the liver [17, 18]. Zeng et al. [19] and Li's [20] researches based on a data mining method have shown that Cuscutae Semen and Herba Taxilli were frequently prescribed for TA treatment and were always used in combination as an herb pair, indicating the key role of these two herbs in TA treatment and the representative value for other akin herbs to explore the potential mechanisms of action on TA treatment. Cuscutae Semen is the dry seed of Cuscuta australis and Cuscuta chinensis, which has been used for varieties of kidney conditions [21]. It has been reported to have neuroprotection properties in mouse models of Parkinson's disease [22] and anti-inflammatory properties [23, 24] and also be effective in treating vitiligo [25]. Herba Taxilli is the dry leafy stem and branch of Taxillus sutchuenensis (Lecomte) Danser which grows on various trees and shrubs [26] and has the anti-HCV [27] and antioxidant activities [28]. Generally speaking, herbal medicines own the features of multicomponents and multieffectiveness in treating diseases which makes it hard to clearly clarify the efficiency in a molecular level. Although Tusizi-Sangjisheng is widely used for the treatment of TA, the exact mechanisms of it remain to be further elucidated.

The objective of this study is to systematically explore the functional mechanism of the herb pair Tusizi-Sangjisheng in the molecular level and also to understand the relationship among those small chemicals, their related targets, and diseases. The results of this study may facilitate clarifying the mechanism of the Chinese herb pair Tusizi-Sangjisheng and offer some valuable methods for studying other akin herbs.

## 2. Materials and Methods

### 2.1. Molecular Database and ADME Screening

All compounds of Tusizi-Sangjisheng in this study were collected by using the TCMSP database (http://lsp.nwsuaf.edu.cn/tcmsp.php) which comprises more than 510 herbal entries registered in Chinese pharmacopoeia with more than 33,000 ingredients. The rational assessment of absorption, distribution, metabolism, and excretion properties (ADME) of compounds is essential to decide drug candidates. In the present study, two ADME-related models, PreOB (predict oral bioavailability) and PreDL (predict drug likeness) of the drugs, were employed to prescreen the bioactive compounds. Oral bioavailability (%*F*) is considered a key parameter in drug development. Oral drug absorption is mainly about two basic parameters: solubility and gastrointestinal permeability of the drug. In detail, the PreOB model, developed on the basis of a robust in-house system OBioavail 1.1 [29], was performed to predict the OB of the constituents of the herbs. The molecules with suitable OB ≥ 30% were chosen as candidate compounds for further research. Drug likeness is a concept that is aimed at identifying where virtual or real molecules fall into drug-like chemical space based on one or more physiochemical properties. According to the mean value (0.18) of DL for all 3,206 molecules in DrugBank (http://www.drugbank.ca/), compounds with DL ≥ 0.18 were selected as the candidate bioactive chemicals in this study. Finally, a total of 75 compounds from Tusizi-Sangjisheng were obtained and 12 bioactive compounds among them were screened.

### 2.2. Target Identification and Network Construction

Target identification for bioactive compounds is an essential step for drug discovery. Predicting drug-target and drug-pathway interactions could help understand the biological mechanisms from the perspective of network pharmacology. To obtain the related targets of these active compounds, a SysDT [30] model based on Random forest (RF) and Support Vector Machine (SVM) was employed. The UniProt database (https://www.uniprot.org/) was used to search the genes of the human species and the corresponding UniProtKB related to the predicted targets of the bioactive molecules. Information on the physiological functions of all targets was obtained from TTD (http://bidd.nus.edu.sg/group/ttd/) and UniProt (http://www.uniprot.org/) databases. Additionally, to systematically study the effects of herbal medicines and to characterize the therapeutic effects on a pathway level, three visualized networks including Compound-Target (C-T), Target-Disease (T-D), and Target-Pathway (T-P) were constructed. And the obtained target profiles were organized into several pathways by mapping to KEGG (Kyoto Encyclopedia of Genes and Genomes). All bipartite graphs were drawn by Cytoscape 3.5.1 software [31].

## 3. Results

### 3.1. Potential Active Compounds

In this study, a total of 75 compounds of the Tusizi-Sangjisheng (29 for Tusizi and 46 for Sangjisheng) were included. After screening all compounds at the criteria that OB ≥ 30% and DL ≥ 0.18, 12 bioactive compounds with excellent ADME properties were obtained and the specific information is shown in Table 1.

### 3.2. Drug Targeting and Network Analysis

Traditionally, herbal medicines contain numerous pharmacological compounds, which offer bright prospects for the treatment of complex diseases in a synergistic manner. Network pharmacology has undergone a rapid development in recent years and emerged as an invaluable tool for describing and analyzing complex systems in pharmacology studies. Considering that the multicomponent herbs exert effects on diseases by acting to specific protein targets, drug targeting may shed light on the mechanism of herbs treating TA from the perspective of network pharmacology. In this study, after target fishing by the SysDT [30] model, a total of 153 targets were screened which were targeted by 10 bioactive compounds (two of the 12 bioactive compounds had no direct targets) from Tusizi-Sangjisheng. The detailed information is presented in Table 2.

#### 3.2.1. Compound-Target Network

**Figure 1:**
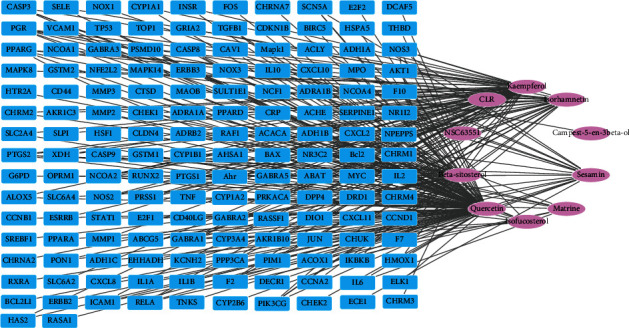
Compound-Target network (C-T). The blue squares represent 153 targets related to active compounds, and the pink circles represent the 10 bioactive compounds.

In order to uncover the synergistic effects of multiple components and targets in Tusizi-Sangjisheng, a C-T network analysis was carried out. After excluding 2 bioactive compounds which have no directly related targets, a graph of C-T interactions was drawn (Figure 1) using 10 bioactive compounds and 153 related targets. As shown in Figure 1, the blue squares represent bioactive molecules in Tusizi-Sangjisheng, and pink circles represent the corresponding targets. Among the 10 compounds, quercetin owns the highest degree (degree = 108), followed by kaempferol, beta-sitosterol, and isorhamnetin, indicating the key roles of these chemicals in Tusizi-Sangjisheng. Targets like prostaglandin G/H synthase 1 (PTGS1), nitric oxide synthase (NOS), tumor necrosis factor (TNF), interleukins-6 (IL-6), and caspase have a high degree of connectivity, revealing the potential functions of treating TA.

#### 3.2.2. Target-Disease Network

Disease-related genes interact in their products and expression patterns. Disease and disease genes are linked together to form a network through known correlations. A variety of disease network diagrams can be generated from the interdependence of cell networks based on human diseases. In these figures, if the phenotype of a disease is related to a molecule, different phenotypes will be linked [32]. In addition, disorders of human diseases should be viewed as disorders of highly connected networks within cells. These new advances and understandings provide a platform for studying the relationship between known genes and diseases, which means that different diseases may originate from the same gene [33].

Existing data and theories make it possible to use network methods to study diseases from different scales. Complex network systems can visually reveal the complex relationships among diseases, genes, and pathways to a certain extent. Since the application of network models to biology, many researchers have constructed networks of different functions and properties. These network models have shown us their value in biological researches. At present, the research on diseases mainly includes protein-protein relationship networks, metabolic networks, and regulatory networks. We established a Target-Disease network to study protein-protein interactions and to understand the complex mechanism of action of TCM. This network showed that different diseases have common pathological changes and can be cured by the same compound; that is, one compound can correspond to multiple diseases.

**Figure 2:**
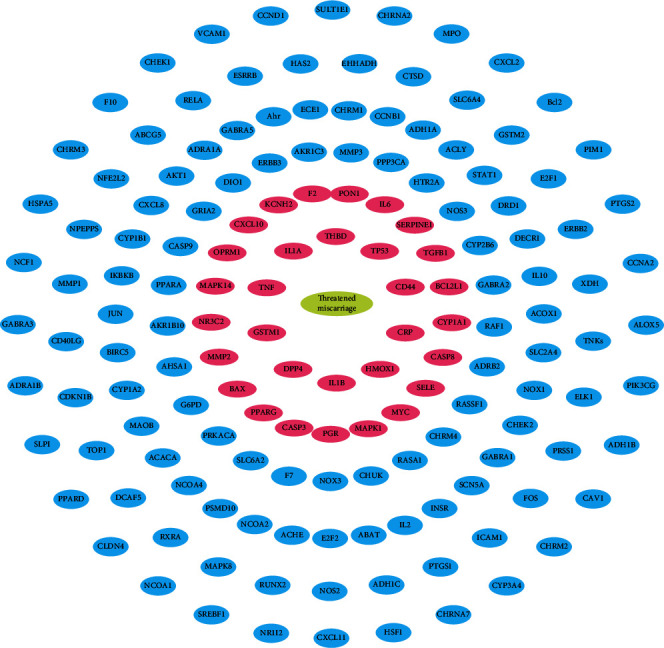
Target-Disease network (T-D). The outer blue circles represent targets related to Tusizi-Sangjisheng, and the inner pink circles represent the targets of TA.

The OMIM database (http://www.omim.org/) was used to search target genes related to TA, and the search term was “threatened miscarriage” or “threatened abortion”. The target genes corresponding to the disease is compared with those corresponding to the bioactive compounds, and the common ones are the target genes of the drug in treating TA. A total of 455 targets related to TA were collected through the OMIM database. Comparing it with the 153 targets corresponding to the bioactive compounds of Tusizi-Sangjisheng, 31 common targets preventing miscarriage were obtained. As shown in Figure 2, the outer blue circles represent the targets of Tusizi-Sangjisheng and the inner pink circles represent the targets of TA.

#### 3.2.3. Target-Pathway Network

**Figure 3:**
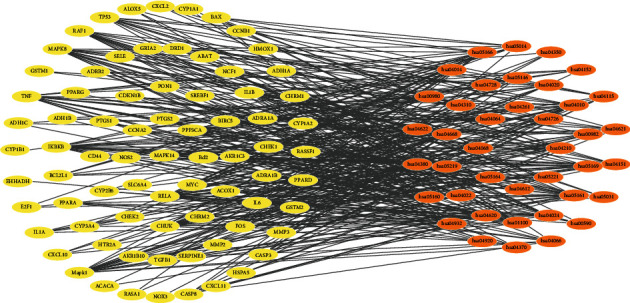
Target-Pathway network (T-P).

As concerns the effects of drugs that are related to target proteins and also signaling pathways associated with diseases, Target-Pathway network was drawn to analyze the action mechanism of Tusizi-Sangjisheng. As shown in Figure 3, the network consists of 116 nodes and 381 edges, and all proteins are involved in more than one signal pathway correlation, forming a highly interconnected network.

### 3.3. GO Analysis

Upload the predicted target genes to the DAVID database (Version 6.8, https://david.ncifcrf.gov/) and perform GO enrichment analysis. We found that these targets are closely related to various biological processes, such as the RNA polymerase II promoter positive regulation of transcription, positive regulation of reactive oxygen metabolic processes, and inflammatory responses to multicellular biological processes.

### 3.4. Pathway Analysis

**Figure 4:**
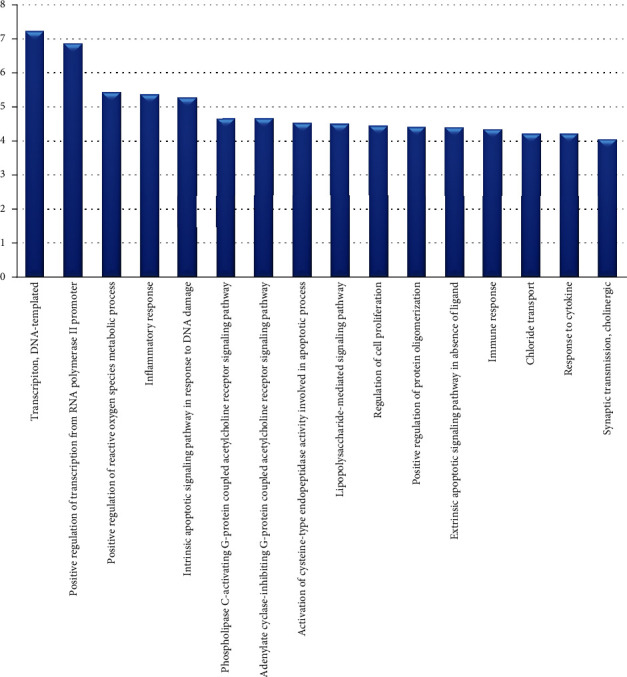
Gene Ontology (GO) analysis of target genes for treatment. The *y*-axis shows a significantly rich “biological process” category in the GO of the target gene, and the *x*-axis shows the enrichment score of these terms.

**Figure 5:**
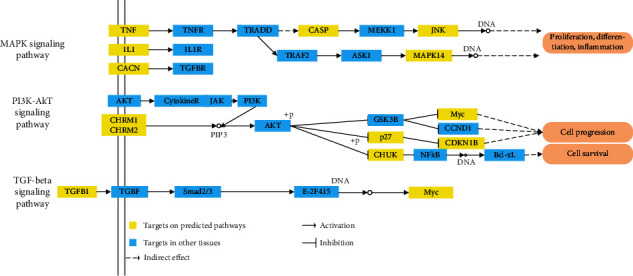
Distribution of the target proteins on compressed TA pathways.

The top three pathways with the highest degrees in Figure 4 are mitogen-activated protein kinase (MAPK) signaling pathway, the phosphatidylinositol-3-kinase/protein kinase B (PI3K-Akt) signaling pathway, and the transforming growth factor-*β* (TGF-*β*) signal transduction pathway. As shown in Figure 5, the MAPK signal pathway is related to targets TNF, IL1, CACN, CASP, JNK, and MAPK14 and exerts the functions of proliferation, differentiation, and inflammation. Up to six targets are involved in the PI3K-Akt pathway as important regulators for the cell progression and survival, including CHRM1, CHRM2, p27, CHUK, Myc, and CDK2. And the TGF-*β* signal pathway acts on TGFB1 and Myc targets.

## 4. Discussions

### 4.1. Bioactive Compounds of the Tusizi-Sangjisheng Herb Pair

In this study, we used systematic pharmacology to study Tusizi-Sangjisheng and found that this herb pair has a total of 12 compounds with good OB and DL properties, which are related to 153 target proteins. The most potent compounds in Tusizi-Sangjisheng is quercetin which is followed by kaempferol, *β*-sitosterol, and isorhamnetin. Quercetin, kaempferol, and isorhamnetin are all flavonoids that are abundant in amount and widely distributed in nature. Quercetin is one of the phytochemicals with anticancer and antioxidant activities that widely exists in nuts, teas, vegetables, herbs, and generally diet of people [34, 35]. In addition to the above effects, quercetin still has the anti-inflammatory functions by blocking the secretion of IL-6, IFN-*γ*, and TNF-*α* and increasing the decreased ratio of Bcl-2/Bax apoptotic proteins induced by lipopolysaccharide (LPS) revealing the putative value in preventing miscarriage caused by bacterial infection [36–38]. Kaempferol is a typical natural flavonol and has been reported to own many beneficial functions such as anti-inflammatory, antioxidative, antiatherogenic, hepatoprotective, neuroprotective, antidiabetic, and anticancer activities [39, 40]. It is well known for its prominent antioxidative activity [39], and the main mechanisms include decreasing the susceptibility of low-density lipoproteins (LDL) to oxidation, inhibiting the release of cytochrome C, scavenging excessive reactive oxygen species (ROS), and inhibiting the generation of ROS by regulating the level of NOS/NO, reducing the accumulation of toxic lipid peroxidation product malondialdehyde (MDA), and maintaining the activities of superoxide dismutase (SOD) and glutathione peroxidase (GPx) in a normal level [41–43]. Isorhamnetin is a flavonoid present in many plants and has been reported to protect against inflammatory and oxidative stress responses in various in vitro and in vivo models using LPS, inflammatory cytokines, and ischemic injury. The anti-inflammatory function of isorhamnetin is regarded to be related to inhibition of NF-*κ*B signaling activity, and its antioxidative effect is associated with ROS blocking [44]. Besides, isorhamnetin also has the anticancer activity by acting on Akt and MAPK signal pathways [45]. *β*-Sitosterol, one of the several phytosterols, is a natural micronutrient in higher plants possessing numerous physiological effects including antioxidative, anticancer, anti-inflammatory, antidiabetic, and immune modulation activities [46]. Although quercetin, kaempferol, isorhamnetin, and *β*-sitosterol have wide pharmacological functions, the detailed correlation with TA is still unclear and needs to be further studied.

### 4.2. Main Targets of the Tusizi-Sangjisheng Herb Pair

#### 4.2.1. PTGS

PTGS, also known as cyclooxygenase (COX) [47], is a crucial enzyme for the synthesis of prostaglandins (PG) in the body, which can transform arachidonic acid into various types of PGs. There are three known subtypes of PTGS: PTGS1, PTGS2, and PTGS3. PTGS1 exists in most cells and exerts the function of maintaining and regulating the normal physiological activities by synthesizing PG. PG is closely related to physiological processes such as endometrial vascular regeneration, vascular permeability, and establishment of a placental vascular network during pregnancy [48]. COX is considered a key factor in implantation [49]. Studies have shown that the expression level of COX-2 in the women's endometrium during the midluteal phase of women's menstrual cycles begins to create the necessary conditions for conception. Once trophoblast invasion occurs, the COX-2 content at the invaded site will increase continuously [50], and IL-1 was found to increase COX-2 expression in endometrial cells at the implantation site [51]. The expression of COX in patients with TA/recurrent miscarriage is lower than that in normal pregnancy, which may interfere the process of embryo implantation through various PG molecules it catalyzes and participate in the pathogenic process of TA.

#### 4.2.2. NOS

NOS is the only rate-limiting enzyme that synthesizes NO. It belongs to isozymes and has three subtypes: neuronal (nNOS/NOS1), inducible (iNOS/NOS2), and endothelial NOS (eNOS/NOS3) [52], among which NOS3 is mainly expressed in vascular endothelial cells and catalyzes the production of intravascular NO. NO is a signaling molecule in the cardiovascular system that has the properties of smooth muscle relaxation, platelet inhibition, leukocyte aggregation, and also attenuation of vascular smooth muscle cell proliferation, neurotransmission, and immune defense [53]. NOS3 is also expressed in the placental tissue during pregnancy. It promotes the synthesis of NO to participate in the regulation of blood circulation in the placenta, causing a slight decrease in vascular resistance and blood pressure, which is of great significance for maintaining uterine placental blood flow. Studies have found that the expression of NOS in endothelial cells in the plasma and placenta is increased in women with preeclampsia of its compensatory mechanisms [54, 55]. The hypothesis of the pathogenesis of preeclampsia is that the expression of NOS decreased and NO synthesis reduced, resulting in increased vascular bed resistance and blocked placental blood circulation, while the successful pregnancy during the first trimester depends on the favoring invasion of trophoblasts to the endometrium and the success remodeling of the uterine spiral arteries, and NO plays important roles in this process. Paradisi et al.'s research has shown that serum NO levels in patients with TA are lower than those in normal pregnancy [56], indicating that TA may be associated with decreased NOS expression which is similar to that of preeclampsia. A reduced NO level results in the disturbance of maternal-fetal circulation and further influences the supply of blood and oxygen which may cause TA.

#### 4.2.3. TNF

TNF is a cytokine secreted by macrophages that can directly cause death of tumor cells. TNF-*α* and TNF-*β* are the two known types of TNF that can be expressed in immune cells such as T and B cells. IL-6 (interleukin 6), which belongs to the Th2-type cytokine, is a type of cytokine involved in a variety of immune inflammatory responses that could inhibit Th1-mediated immune responses. Th1 immunity causes loss of pregnancy while Th2 immunity helps to maintain pregnancy, so the ratio of Th1/Th2 is crucial for pregnancy. If the balance of Th1/Th2 is broken, TA or miscarriage may occur. TNF-*α* is mainly expressed by Th1 and NK cells that can kill tumor cells and also attack trophoblast cells and embryonic tissues. A high serum level of TNF-*α* and inadequate expression of IL-6 in endometrial tissue have been found in patients with recurrent abortion, revealing that the overproduction of TNF or underactivation of IL-6 may cause fetus damage and loss [57, 58]. In this study, the active ingredients in Tusizi-Sangjisheng act on the targets IL-6 and TNF-*α* to promote the expression of IL-6, increase its level in plasma and decidual tissue, and then inhibit the expression of TNF-*α* so as to reduce the maternal immune response against the fetus and maintain the normal development of the pregnancy.

#### 4.2.4. Caspase

Caspase is a type of protease and an important mediator of programmed cell death (apoptosis) that includes caspase-1 (containing caspase-1, 4, 5, and 11), caspase-2 (containing caspase-2 and 9), and caspase-3 (containing caspase-3, 6, 7, 8, and 10). Among them, caspase-3 is mainly expressed in the cytoplasm of trophoblast cells and could regulate cell growth and apoptosis by activating the Fas pathway. Apoptosis is essential for the normal physiology of pregnancy during implantation since apoptosis of trophoblast cells is important for the appropriate tissue remodeling of the maternal decidua and invasion of the developing embryo [59, 60]. The overexpression of caspase-3 will cause abnormal apoptosis of trophoblasts, affect embryo development, and result in abortion. Meresman et al.'s study indicated that the expression level of caspase-3 in out-of-phase endometrium abortion patients is higher than that in normal, and the decreased cell proliferation and augmented cell apoptosis were also found [61]. The bioactive components of Tusizi-Sangjisheng may act on caspase-3 and suppress its expression to reduce the apoptosis of trophoblast cells.

### 4.3. Signal Pathways of the Tusizi-Sangjisheng Herb Pair

#### 4.3.1. MAPK Signal Pathway

MAPK is a class of serine/threonine protein kinases and widely present in various cells. It has important regulatory effects on gene expression. After activation, the protein migrates into the phosphorylated nucleoproteins and membrane receptors, regulating gene transcription and other life events. It consists of four separate signaling cascades: the JNK/SAPK (c-Jun N-terminal kinase/stress-activated protein kinases); the ERKs (extracellular signal-regulated kinases); the ERK5 or big MAPK1; and the p38MAPK group of protein kinases. When stimulated by extracellular signals, the MAPK cascade which is an evolutionarily conserved tertiary kinase cascade transduction signal will be activated by a series of complicated chemical reactions, resulting in the successive activation of MAPKKK, MAPKK, and MAPK through the phosphorylation of amino acid residues [62]. Evidences have shown that the MAPK signal pathway is activated in many processes during embryo implantation and is closely related to the invasion and proliferation of trophoblast cells and decidual stromal cells (DSCs) which play key roles in the maintenance of normal pregnancy [63, 64]. Z. Wang et al.'s research demonstrated the abnormal expressions of nucleotide-binding oligomerization domains 1 and 2 (NOD 1 and NOD 2) in villi from recurrent spontaneous abortion (RSA) patients that then inhibit the invasion and proliferation of trophoblast cells by activating the p38MAPK signal pathway [65]. In addition, inflammation (including sterile inflammation or infectious inflammation induced by bacteria such as bacterial vaginosis) and oxidative stress (smoking) could also activate the p38MAPK signal pathway and thus attenuate the normal development of trophoblast cells [66]. Furthermore, the activation of the JNK and ERK1/2 signal pathway by interleukin (IL)-33 [67] and IL-25 [68] also leads to the enhancement of invasion and proliferation of DSCs. Accordingly, for the similar pathogenesis between RAS and TA, the role of the MAPK signal pathway could also be considered by affecting the invasion and proliferation of trophoblast cells and DSCs even though there is no direct evidence. The total flavones of Cuscutae Semen were found to be effective in suppressing abortion by inhibiting MAPK pathways [69], indicating the potential mechanism of the herb pair Tusizi-Sangjisheng treating TA.

#### 4.3.2. PI3K-Akt Signal Pathway

The PI3K-Akt signal pathway consists of phosphoinositide 3-kinase (PI3K) and protein kinase B (PKB, also known as Akt) and is involved in transcription, protein synthesis, migration, apoptosis, and proliferation activities [70]. Identically, the PI3K-Akt signaling pathway also participates in the proliferation and migration of trophoblast cells that are vital for establishing and persisting normal success pregnancy. Evidence has shown that the up-/downregulation of the PI3K-Akt signal pathway is related to the proliferation and migration of trophoblast cells [71]. PI3K signaling is considered to have association with the secretion of several hormones including gonadotropin-releasing hormone (GnRH)/luteinizing hormone (LH) that are important in the maintenance of normal pregnancy [72]. In addition, PI3K could also regulate insulin signaling via activating the Akt pathway [72]. Generally, obese persons with insulin resistance are often companied by reduced hypothalamic GnRH secretion and decreased fertility [73]. An experimental study also demonstrated that herbal decoctions containing herbs prevent miscarriage and are effective to promote the follicle development and fertility via activation of the PI3K-Akt signal pathway [74]. Accordingly, we assume that Tusizi-Sangjisheng may suppress miscarriage via the PI3K-Akt signal pathway by influencing the expression of sex hormones GnRH/LH and also the proliferation and migration of trophoblast cells.

#### 4.3.3. TGF-*β* Signal Pathway

TGF-*β* is a multifunctional cytokine with three isoforms (TGF-*β*1, TGF-*β*2, and TGF-*β*3) and acts mainly through Smad pathways [75]. Evidences have proved that TGF-*β* plays pivotal and dual roles in the regulation of cell growth and apoptosis which is vividly described as a “switch” [76]. During early pregnancy, the uterine cells undergo apoptosis and proliferation for the successful implantation of embryo. Those complex processes are controlled and regulated by several signal pathways, and the TGF-*β* signal pathway is one of them that could help to maintain normal pregnancy by balancing the proliferation and apoptosis of trophoblast cells [75]. In addition, the TGF-*β* signal pathway is also involved in immune and inflammatory responses by regulating indoleamine 2,3-dioxygense (IDO) expression to promote immune tolerance of the maternal-fetal interface [77], inhibiting the proliferation of human Th1 memory T cells [78]. At present, there is no direct evidence illustrating the specific relationship among herbs like Cuscutae Semen and the TGF-*β* signal pathway in TA. Yet we can hypothesize based on the above information that the active ingredients in the herb pair Tusizi-Sangjisheng exert their TA-preventing function by regulating the immune responses in maternal-fetal interface and controlling the normal apoptosis and proliferation of uterine cells through the TGF-*β* signal pathway.

In normal pregnancy, the balance of the immune microenvironment at the maternal-fetal interface can ensure immune tolerance and protect the embryo from maternal immune rejection. The ability of gestational trophoblasts to proliferate, differentiate, migrate, and invade is crucial for embryo implantation, placenta formation, and embryo growth and development. Trophoblasts are the main cell types of placental tissues that differentiate into extravillous trophoblasts, syncytiotrophoblasts and cell trophoblasts. Extravillous trophoblasts can be further differentiated into cells with high infiltration capacity. Interstitial trophoblast cells and intravascular trophoblast cells then invade the uterine decidua and reshape the spiral arteries of a pregnant uterus, thereby forming placental tissues which provide nutrients for the development of the embryo. Therefore, infiltration of trophoblasts into the uterine decidua is the key step to the success of early pregnancy. If the ability of trophoblasts to proliferate, migrate, and invade is weakened, it will affect the remodeling of decidual tissue blood vessels and the formation of placenta and further develop into pregnancy-dependent complications such as abortion, preeclampsia, and intrauterine growth restriction. These processes during pregnancy are jointly regulated by many signal transduction pathways. However, the exact regulatory mechanisms are still not very clear. Three key signaling pathways mentioned above were screened by systematic methods in this study revealing that Tusizi-Sangjisheng may effectively reduce miscarriage rates via regulating the normal expression and function of those three signal pathways, correcting under- or overexpression and maintaining normal pregnancy.

## 5. Conclusions

The putative mechanisms in the molecular level of the commonly used herb pair Tusizi-Sangjisheng treating TA were analyzed by systems pharmacology. 12 bioactive molecules with OB ≥ 30% and DL ≥ 0.18 of Tusizi-Sangjisheng were obtained by calculating the ADME properties. Besides, a total of 153 direct targets of 12 bioactive molecules were predicted by systematic models. By constructing a T-D network for analysis, 31 targets mainly containing PTGS1, NOS3, TNF, and caspase were found to be directly related to TA among the corresponding targets of Tusizi-Sangjisheng and TA was considered a complex disease with multifaceted causes that need to be treated from multiple angles by GO analysis. Through the T-P network enrichment analysis, Tusizi-Sangjisheng was found to act on multiple pathways by several active molecules that include MAPK, PI3K-Akt, and TGF-*β* signaling pathways. The mechanism of Tusizi-Sangjisheng on the treatment of TA may be through regulating the normal expression of the above three signal pathways, correcting the state of under- or overexpression, and maintaining normal pregnancy.

## Figures and Tables

**Table 1:** Bioactive compounds of Cuscutae Semen and Herba Taxilli.

ID	Compound	OB	DL	Herb name
MOL001558	Sesamin	56.55	0.83	Cuscutae Semen
MOL000184	NSC63551	39.25	0.76	Cuscutae Semen
MOL000098	Quercetin	46.43	0.28	Herba Taxilli/Cuscutae Semen
MOL000354	Isorhamnetin	49.60	0.31	Cuscutae Semen
MOL000358	Beta-sitosterol	36.91	0.75	Cuscutae Semen
MOL000422	Kaempferol	41.88	0.24	Cuscutae Semen
MOL000953	CLR	37.87	0.68	Cuscutae Semen
MOL005043	Campest-5-en-3beta-ol	37.58	0.71	Cuscutae Semen
MOL005440	Isofucosterol	43.78	0.76	Cuscutae Semen
MOL005944	Matrine	63.77	0.25	Cuscutae Semen
MOL006649	Sophranol	55.42	0.28	Cuscutae Semen
MOL000359	Sitosterol	36.91	0.75	Herba Taxilli

**Table 2:** The information of related targets of the Tusizi-Sangjisheng herb pair.

No.	Target name	Gene name	UniProt ID
1	26S proteasome non-ATPase regulatory subunit 3	PSMD10	O75832
2	72 kDa type IV collagenase	MMP2	P08253
3	78 kDa glucose-regulated protein	HSPA5	P11021
4	Acetylcholinesterase	ACHE	P22303
5	Activator of 90 kDa heat shock protein ATPase homolog 1	AHSA1	O95433
6	Androgen receptor	NCOA4	Q13772
7	Apoptosis regulator BAX	BAX	Q07812
8	Arachidonate 5-lipoxygenase	ALOX5	P09917
9	ATP-binding cassette subfamily G member 2	ABCG5	Q9H222
10	Baculoviral IAP repeat-containing protein 5	BIRC5	O15392
11	Bcl-2-like protein 1	BCL2L1	Q07817
12	Beta-2 adrenergic receptor	ADRB2	P07550
13	Caspase-8	CASP8	Q14790
14	Cathepsin D	CTSD	P07339
15	Caveolin-1	CAV1	Q03135
16	CD40 ligand	CD40LG	P29965
17	Cellular tumor antigen p53	TP53	P04637
18	Claudin-4	CLDN4	O14493
19	Coagulation factor VII	F7	P08709
20	C-reactive protein	CRP	P02741
21	C-X-C motif chemokine 10	CXCL10	P02778
22	C-X-C motif chemokine 11	CXCL11	O14625
23	C-X-C motif chemokine 2	CXCL2	P19875
24	Cyclin-dependent kinase inhibitor 1	CDKN1B	P46527
25	Cytochrome P450 1A1	CYP1A1	P04798
26	Cytochrome P450 1B1	CYP1B1	Q16678
27	Cytochrome P450 3A4	CYP3A4	P08684
28	DDB1- and CUL4-associated factor 5	DCAF5	Q96JK2
29	Dipeptidyl peptidase IV	DPP4	P27487
30	DNA topoisomerase 1	TOP1	P11387
31	E-selectin	SELE	P16581
32	Estrogen sulfotransferase	SULT1E1	P49888
33	ETS domain-containing protein Elk-1	ELK1	P19419
34	G1/S-specific cyclin-D1	CCND1	P24385
35	G2/mitotic-specific cyclin-B1	CCNB1	P14635
36	Gamma-aminobutyric acid receptor subunit alpha-1	GABRA1	P14867
37	Glutathione S-transferase Mu 1	GSTM1	P09488
38	Heat shock factor protein 1	HSF1	Q00613
39	Heme oxygenase 1	HMOX1	P09601
40	Inhibitor of nuclear factor kappa-B kinase subunit alpha	CHUK	O15111
41	Insulin receptor	INSR	P06213
42	Interleukin-1 alpha	IL1A	P01583
43	Interleukin-1 beta	IL1B	P01584
44	Interleukin-10	IL10	P22301
45	Interleukin-2	IL2	P60568
46	Interleukin-6	IL6	P05231
47	Interleukin-8	CXCL8	P10145
48	Interstitial collagenase	MMP1	P03956
49	Mitogen-activated protein kinase 1	Mapk1	P63085
50	mRNA of PKA catalytic subunit C-alpha	PRKACA	P17612
51	Myc protooncogene protein	MYC	P01106
52	Myeloperoxidase	MPO	P05164
53	Neutrophil cytosol factor 1	NCF1	P14598
54	Nuclear factor erythroid 2-related factor 2	NFE2L2	Q16236
55	Nuclear receptor coactivator 2	NCOA2	Q15596
56	Peroxisome proliferator-activated receptor alpha	PPARA	Q07869
57	Peroxisome proliferator activated receptor delta	PPARD	Q03181
58	Peroxisome proliferator activated receptor gamma	PPARG	P37231
59	Plasminogen activator inhibitor 1	SERPINE1	P05121
60	Poly [ADP-ribose] polymerase 1	TNKS	O95271
61	Potassium voltage-gated channel subfamily H member 2	KCNH2	Q12809
62	Prostaglandin G/H synthase 1	PTGS1	P23219
63	Protooncogene c-Fos	FOS	P01100
64	Puromycin-sensitive aminopeptidase	NPEPPS	P55786
65	RAC-alpha serine/threonine-protein kinase	AKT1	P31749
66	RAF protooncogene serine/threonine-protein kinase	RAF1	P04049
67	Ras association domain-containing protein 1	RASSF1	Q9NS23
68	Ras GTPase-activating protein 1	RASA1	P20936
69	Receptor tyrosine-protein kinase erbB-2	ERBB2	P04626
70	Receptor tyrosine-protein kinase erbB-3	ERBB3	P21860
71	Retinoic acid receptor RXR-alpha	RXRA	P19793
72	Runt-related transcription factor 2	RUNX2	Q13950
73	Serine/threonine-protein kinase Chk2	CHEK2	O96017
74	Serum paraoxonase/arylesterase 1	PON1	P27169
75	Sodium channel protein type 5 subunit alpha	SCN5A	Q14524
76	Solute carrier family 2, facilitated glucose transporter member 4	SLC2A4	P14672
77	Stromelysin-1	MMP3	P08254
78	Thrombin	F2	P00734
79	Thrombomodulin	THBD	P07204
80	Transcription factor AP-1	JUN	P05412
81	Transcription factor E2F1	E2F1	Q01094
82	Transcription factor E2F2	E2F2	Q14209
83	Transcription factor p65	RELA	Q04206
84	Trypsin-1	PRSS1	P07477
85	Type I iodothyronine deiodinase	DIO1	P49895
86	Xanthine dehydrogenase/oxidase	XDH	P47989
87	2,4-Dienoyl-CoA reductase, mitochondrial	DECR1	Q16698
88	Acetyl-CoA carboxylase 1	ACACA	Q13085
89	ATP-citrate synthase	ACLY	P53396
90	Coagulation factor Xa	F10	P00742
91	Cytochrome P450 2B6	CYP2B6	P20813
92	Endothelin-converting enzyme 1	ECE1	P42892
93	Glucose-6-phosphate 1-dehydrogenase	G6PD	P11413
94	NADPH oxidase 1	NOX1	Q9Y5S8
95	NADPH oxidase 3	NOX3	Q9HBY0
96	Nitric oxide synthase, endothelial	NOS3	P29474
97	Peroxisomal acyl-coenzyme A oxidase 1	ACOX1	Q15067
98	Peroxisomal bifunctional enzyme	EHHADH	Q08426
99	Sterol regulatory element-binding protein 1	SREBF1	P36956
100	Aldose reductase	AKR1B10	O60218
101	Amine oxidase [flavin-containing] B	MAOB	P27338
102	Cyclin-A2	CCNA2	P20248
103	Estrogen receptor beta	ESRRB	O95718
104	Glutamate receptor 2	GRIA2	P42262
105	Mitogen-activated protein kinase 14	MAPK14	Q16539
106	Nitric oxide synthase, inducible	NOS2	P35228
107	Nuclear receptor coactivator 1	NCOA1	Q15788
108	Protooncogene serine/threonine-protein kinase Pim-1	PIM1	P11309
109	Serine/threonine-protein kinase Chk1	CHEK1	O14757
110	5-Hydroxytryptamine 2A receptor	HTR2A	P28223
111	Alpha-1A adrenergic receptor	ADRA1A	P35348
112	Alpha-1B adrenergic receptor	ADRA1B	P35368
113	Caspase-9	CASP9	P55211
114	Dopamine D1 receptor	DRD1	Q95136
115	Gamma-aminobutyric-acid receptor alpha-3 subunit	GABRA3	P34903
116	Gamma-aminobutyric-acid receptor alpha-5 subunit	GABRA5	P31644
117	Muscarinic acetylcholine receptor M2	CHRM2	P08172
118	Muscarinic acetylcholine receptor M3	CHRM3	P20309
119	Muscarinic acetylcholine receptor M4	CHRM4	P08173
120	Mu-type opioid receptor	OPRM1	P35372
121	Neuronal acetylcholine receptor protein, alpha-7 chain	CHRNA7	P36544
122	Neuronal acetylcholine receptor subunit alpha-2	CHRNA2	Q15822
123	Sodium-dependent serotonin transporter	SLC6A4	P31645
124	Transforming growth factor beta-1	TGFB1	P01137
125	Aldo-keto reductase family 1 member C3	AKR1C3	P42330
126	Antileukoproteinase	SLPI	P03973
127	Apoptosis regulator Bcl-2	Bcl2	P10417
128	Aryl hydrocarbon receptor	Ahr	P30561
129	Cytochrome P450 1A2	CYP1A2	P05177
130	Gamma-aminobutyric-acid receptor alpha-2 subunit	GABRA2	P47869
131	Glutathione S-transferase Mu 2	GSTM2	P28161
132	Hyaluronan synthase 2	HAS2	Q92819
133	Inhibitor of nuclear factor kappa-B kinase subunit beta	IKBKB	O14920
134	Mitogen-activated protein kinase 8	MAPK8	P45983
135	Muscarinic acetylcholine receptor M1	CHRM1	P11229
136	Nitric oxide synthase, endothelial	NOS3	P29474
137	Nuclear receptor subfamily 1 group I member 2	NR1I2	O75469
138	Phosphatidylinositol-4,5-bisphosphate 3-kinase catalytic subunit, gamma isoform	PIK3CG	P48736
139	Prostaglandin G/H synthase 2	PTGS2	P35354
140	Serine/threonine-protein phosphatase 2B catalytic subunit alpha isoform	PPP3CA	Q08209
141	Signal transducer and activator of transcription 1-alpha/beta	STAT1	P42224
142	Sodium-dependent noradrenaline transporter	SLC6A2	P23975
143	Vascular cell adhesion protein 1	VCAM1	P19320
144	4-Aminobutyrate aminotransferase, mitochondrial	ABAT	P80404
145	Alcohol dehydrogenase 1A	ADH1A	P07327
146	Alcohol dehydrogenase 1B	ADH1B	P00325
147	Alcohol dehydrogenase 1C	ADH1C	P00326
148	Caspase-3	CASP3	P42574
149	CD44 antigen	CD44	P16070
150	Intercellular adhesion molecule 1	ICAM1	P05362
151	Tumor necrosis factor	TNF	P01375
152	Mineralocorticoid receptor	NR3C2	P08235
153	Progesterone receptor	PGR	P06401

## Data Availability

The data used to support the findings of this study are included in the article.

## References

[1] Makrydimas G., Sebire N. J., Lolis D., Vlassis N., Nicolaides K. H. (2003). Fetal loss following ultrasound diagnosis of a live fetus at 6-10 weeks of gestation. *Ultrasound in Obstetrics and Gynecology*.

[2] Sotiriadis A., Papatheodorou S., Makrydimas G. (2004). Threatened miscarriage: evaluation and management. *BMJ*.

[3] Everett C. (1997). Incidence and outcome of bleeding before the 20th week of pregnancy: prospective study from general practice. *BMJ*.

[4] Carp H. (2015). A systematic review of dydrogesterone for the treatment of recurrent miscarriage. *Gynecological Endocrinology*.

[5] Weiss J. L., Malone F. D., Vidaver J. (2004). Threatened abortion: a risk factor for poor pregnancy outcome, a population- based screening study. *American Journal of Obstetrics and Gynecology*.

[6] Simpson J. L. (2007). Causes of fetal wastage. *Clinical Obstetrics and Gynecology*.

[7] Tien J. C., Tan T. Y. (2007). Non-surgical interventions for threatened and recurrent miscarriages. *Singapore Medical Journal*.

[8] Lede R. L., Duley L. (2005). Uterine muscle relaxant drugs for threatened miscarriage. *Cochrane Database of Systematic Reviews*.

[9] Xu Q., Chen J., Wei Z. (2017). Sex hormone metabolism and threatened abortion. *Medical Science Monitor*.

[10] Stray-Pedersen B., Stray-Pedersen S. (1984). Etiologic factors and subsequent reproductive performance in 195 couples with a prior history of habitual abortion. *American Journal of Obstetrics and Gynecology*.

[11] Aleman A., Althabe F., Belizán J. M., Bergel E. (2005). Bed rest during pregnancy for preventing miscarriage. *Cochrane Database of Systematic Reviews*.

[12] Yuan S., Gao F., Xin Z. (2019). Comparison of the efficacy and safety of phloroglucinol and magnesium sulfate in the treatment of threatened abortion: a meta-analysis of randomized controlled trials. *Medicine*.

[13] Lee H. J., Park T. C., Kim J. H., Norwitz E., Lee B. (2017). The influence of oral dydrogesterone and vaginal progesterone on threatened abortion: a systematic review and meta-analysis. *BioMed Research International*.

[14] Greene M. F. (2019). Progesterone for threatened abortion. *New England Journal of Medicine*.

[15] Lim C. E., Ho K. K., Cheng N. C., Wong F. W. (2013). Combined oestrogen and progesterone for preventing miscarriage. *Cochrane Database of Systematic Reviews*.

[16] Weinberg L. (2001). Use of anti-D immunoglobulin in the treatment of threatened miscarriage in the accident and emergency department. *Emergency Medicine Journal*.

[17] Ding J., Tan X., Song K. (2018). Bushen Huoxue recipe alleviates implantation loss in mice by enhancing estrogen–progesterone signals and promoting decidual angiogenesis through FGF2 during early pregnancy. *Frontiers in Pharmacology*.

[18] Qu F., Zhou J. (2006). Treating threatened abortion with Chinese herbs: a case report. *Phytotherapy research*.

[19] Dan Z., Jing-feng M., Hui T., Wu F.-t., Chao-feng O. (2018). Research on medication rules of traditional Chinese medicine in treating threatened abortion based on association rule. *Chin. Med. J. Res. Prac*.

[20] Li S. (2019). Exploration on the medication rules of threatened abortion of famous-aged Chinese doctors based on traditional Chinese medicine inheritance auxiliary platform. *Beijing university of Chinese medicine*.

[21] Tang J.-L., Liu B.-Y., Ma K.-W. (2008). Traditional Chinese medicine. *The Lancet*.

[22] Ye M., Lee S. g., Chung E. S. (2014). Neuroprotective effects of Cuscutae Semen in a mouse model of Parkinson’s disease. *Evidence-Based Complementary and Alternative Medicine*.

[23] Kang S. Y., Jung H. W., Lee M.-Y., Lee H. W., Chae S. W., Park Y.-K. (2014). Effect of the semen extract of _Cuscuta chinensis_ on inflammatory responses in LPS-stimulated BV-2 microglia. *Chinese Journal of Natural Medicines*.

[24] Liao J.-C., Chang W.-T., Lee M.-S. (2014). Antinociceptive and anti-inflammatory activities of *Cuscuta chinensis* seeds in mice. *The American Journal of Chinese Medicine*.

[25] Yang B., Yang Q., Yang X., Yan H.-B., Lu Q.-P. (2016). Hyperoside protects human primary melanocytes against H2O2-induced oxidative damage. *Molecular Medicine Reports*.

[26] Li M. R., Li L. Q., Li P. (1987). Flavonoids of Taxillus sutchuenensis (Lecomte) Danser and T. sutchuenensis var. duclouxii (Lecomte) Kiuined. *Zhong yao tong bao (Beijing, China: 1981)*.

[27] Yang L., Lin J., Zhou B., Liu Y., Zhu B. (2016). Activity of compounds from Taxillus sutchuenensis as inhibitors of HCV NS3 serine protease. *Natural Product Research*.

[28] Chan S., Li S., Kwok C. (2008). Antioxidant activity of Chinese medicinal herbs. *Pharmaceutical Biology*.

[29] Xu X., Zhang W., Huang C. (2012). A novel chemometric method for the prediction of human oral bioavailability. *International Journal of Molecular Sciences*.

[30] Yu H., Chen J., Xu X. (2012). A systematic prediction of multiple drug-target interactions from chemical, genomic, and pharmacological data. *PLoS One*.

[31] Shannon P., Markiel A., Ozier O. (2003). Cytoscape: a software environment for integrated models of biomolecular interaction networks. *Genome Research*.

[32] Vidal M., Cusick M. E., Barabási A. L. (2011). Interactome networks and human disease. *Cell*.

[33] Goh K. I., Cusick M. E., Valle D., Childs B., Vidal M., Barabási A. L. (2007). The human disease network. *Proceedings of the National Academy of Sciences*.

[34] Metodiewa D., Jaiswal A. K., Cenas N., Dickancaité E., Segura-Aguilar J. (1999). Quercetin may act as a cytotoxic prooxidant after its metabolic activation to semiquinone and quinoidal product. *Free Radical Biology and Medicine*.

[35] Shafabakhsh R., Asemi Z. (2019). Quercetin: a natural compound for ovarian cancer treatment. *Journal of Ovarian Research*.

[36] Chen S., Jiang H., Wu X., Fang J. (2016). Therapeutic effects of quercetin on inflammation, obesity, and type 2 diabetes. *Mediators of Inflammation*.

[37] Li Y., Yao J., Han C. (2016). Quercetin, inflammation and immunity. *Nutrients*.

[38] Xiaodan W., Yongping Y., Liu Y., Mu L., Xiuhui Z. (2016). Effect of quercetin on the expression of Bcl-2/Bax apoptotic proteins in endometrial cells of lipopolysaccharide-induced-abortion mice. *Journal of Traditional Chinese Medicine*.

[39] Deng S.-P., Yang Y.-L., Cheng X.-X., Li W.-R., Cai J.-Y. (2019). Synthesis, spectroscopic study and radical scavenging activity of kaempferol derivatives: enhanced water solubility and antioxidant activity. *International Journal of Molecular Sciences*.

[40] Wang J., Fang X., Ge L. (2018). Antitumor, antioxidant and anti-inflammatory activities of kaempferol and its corresponding glycosides and the enzymatic preparation of kaempferol. *PLoS One*.

[41] Ruiz E., Padilla E., Redondo S., Gordillo-Moscoso A., Tejerina T. (2006). Kaempferol inhibits apoptosis in vascular smooth muscle induced by a component of oxidized LDL. *European Journal of Pharmacology*.

[42] Tu Y. C., Lian T. W., Yen J. H., Chen Z. T., Wu M. J. (2007). Antiatherogenic effects of kaempferol and rhamnocitrin. *Journal of Agricultural and Food Chemistry*.

[43] Liao W., Chen L., Ma X., Jiao R., Li X., Wang Y. (2016). Protective effects of kaempferol against reactive oxygen species-induced hemolysis and its antiproliferative activity on human cancer cells. *European Journal of Medicinal Chemistry*.

[44] Kim S. Y., Jin C., Kim C. H. (2019). Isorhamnetin alleviates lipopolysaccharide-induced inflammatory responses in BV2 microglia by inactivating NF-*κ*B, blocking the TLR4 pathway and reducing ROS generation. *International Journal of Molecular Medicine*.

[45] Hu S., Huang L., Meng L., Sun H., Zhang W., Xu Y. (2015). Isorhamnetin inhibits cell proliferation and induces apoptosis in breast cancer via Akt and mitogen-activated protein kinase kinase signaling pathways. *Molecular Medicine Reports*.

[46] Bin Sayeed M. S., Karim S. M. R., Sharmin T., Morshed M. M. (2016). Critical analysis on characterization, systemic effect, and therapeutic potential of beta-sitosterol: a plant-derived orphan phytosterol. *Medicines*.

[47] Makar K. W., Poole E. M., Resler A. J. (2013). COX-1 (PTGS1) and COX-2 (PTGS2) polymorphisms, NSAID interactions, and risk of colon and rectal cancers in two independent populations. *Cancer Causes & Control*.

[48] Chakraborty I., Das S. K., Wang J., Dey S. K. (1996). Developmental expression of the cyclo-oxygenase-1 and cyclo-oxygenase-2 genes in the peri-implantation mouse uterus and their differential regulation by the blastocyst and ovarian steroids. *Journal of Molecular Endocrinology*.

[49] Shah B. H., Catt K. J. (2005). Roles of LPA3 and COX-2 in implantation. *Trends in Endocrinology & Metabolism.*.

[50] Habenicht A. J., Goerig M., Grulich J. (1985). Human platelet-derived growth factor stimulates prostaglandin synthesis by activation and by rapid de novo synthesis of cyclooxygenase. *Journal of clinical investigation*.

[51] Raz A., Wyche A., Siegel N., Needleman P. (1988). Regulation of fibroblast cyclooxygenase synthesis by interleukin-1. *The Journal of Biological Chemistry*.

[52] NASEEM K. (2005). The role of nitric oxide in cardiovascular diseases. *Molecular Aspects of Medicine*.

[53] Bian K., Doursout M. F., Murad F. (2008). Vascular system: role of nitric oxide in cardiovascular diseases. *The Journal of Clinical Hypertension*.

[54] Davidge S. T., Baker P. N., Roberts J. M. (1995). NOS expression is increased in endothelial cells exposed to plasma from women with preeclampsia. *American Journal of Physiology-Heart and Circulatory Physiology*.

[55] Ghabour M. S., Eis A. L. W., Brockman D. E., Pollock J. S., Myatt L. (1995). Immunohistochemical characterization of placental nitric oxide synthase expression in preeclampsia. *American Journal of Obstetrics and Gynecology*.

[56] Paradisi R., Fabbri R., Battaglia C., Facchinetti F., Venturoli S. (2007). Nitric oxide levels in women with missed and threatened abortion: results of a pilot study. *Fertility and Sterility*.

[57] Arslan E., Çolakoğlu M., Çelik Ç. (2004). Serum TNF-*α*, IL-6, lupus anticoagulant and anticardiolipin antibody in women with and without a past history of recurrent miscarriage. *Archives of Gynecology and Obstetrics*.

[58] Jasper M. J., Tremellen K. P., Robertson S. A. (2007). Reduced expression of IL-6 and IL-1*α* mRNAs in secretory phase endometrium of women with recurrent miscarriage. *Journal of Reproductive Immunology*.

[59] Huppertz B., Hemmings D., Renaud S. J., Bulmer J. N., Dash P., Chamley L. W. (2005). Extravillous trophoblast apoptosis – a workshop report. *Placenta*.

[60] Mor G., Abrahams V. M. (2003). Potential role of macrophages as immunoregulators of pregnancy. *Reproductive biology and endocrinology : RB&E.*.

[61] Meresman G. F., Olivares C., Vighi S., Alfie M., Irigoyen M., Etchepareborda J. J. (2010). Apoptosis is increased and cell proliferation is decreased in out-of-phase endometria from infertile and recurrent abortion patients. *Reproductive biology and endocrinology : RB&E.*.

[62] Pearson G., Robinson F., Beers Gibson T. (2001). Mitogen-activated protein (MAP) kinase pathways: regulation and physiological functions. *Endocrine Reviews*.

[63] Paliga A. J. M., Natale D. R., Watson A. J. (2005). p38 mitogen-activated protein kinase (MAPK) first regulates filamentous actin at the 8-16-cell stage during preimplantation development. *Biology of the Cell*.

[64] Tebar F., Villalonga P., Sorkina T., Agell N., Sorkin A., Enrich C. (2002). Calmodulin regulates intracellular trafficking of epidermal growth factor receptor and the MAPK signaling pathway. *Molecular Biology of the Cell*.

[65] Wang Z., Liu M., Nie X. (2015). NOD1 and NOD2 control the invasiveness of trophoblast cells via the MAPK/p38 signaling pathway in human first-trimester pregnancy. *Placenta*.

[66] Menon R., Papaconstantinou J. (2016). p38 mitogen activated protein kinase (MAPK): a new therapeutic target for reducing the risk of adverse pregnancy outcomes. *Expert Opinion on Therapeutic Targets*.

[67] Hu W. T., Li M. Q., Liu W., Jin L. P., Li D. J., Zhu X. Y. (2014). IL-33 enhances proliferation and invasiveness of decidual stromal cells by up-regulation of CCL2/CCR2 via NF-*κ*B and ERK1/2 signaling. *Molecular Human Reproduction*.

[68] Wang Y., Zhang Y., Li M. Q. (2014). Interleukin-25 induced by human chorionic gonadotropin promotes the proliferation of decidual stromal cells by activation of JNK and AKT signal pathways. *Fertility and Sterility*.

[69] Wu H. W., Feng Y. H., Wang D. Y. (2018). Effect of total flavones from Cuscuta chinensis on anti-abortion via the MAPK signaling pathway. *Evidence Based Complementary and Alternative Medicine*.

[70] Yang B., Song J., Sun H. (2018). PSMB8 regulates glioma cell migration, proliferation, and apoptosis through modulating ERK1/2 and PI3K/AKT signaling pathways. *Biomedicine & Pharmacotherapy*.

[71] Li Z., Zhou G., Jiang L., Xiang H., Cao Y. (2018). Effect of STOX1 on recurrent spontaneous abortion by regulating trophoblast cell proliferation and migration via the PI3K/AKT signaling pathway. *Journal of Cellular Biochemistry*.

[72] Acosta-Martínez M. (2012). PI3K: an attractive candidate for the central integration of metabolism and reproduction. *Frontiers in Endocrinology*.

[73] Brothers K. J., Wu S., DiVall S. A. (2010). Rescue of Obesity-Induced Infertility in Female Mice due to a Pituitary- Specific Knockout of the Insulin Receptor. *Cell Metabolism*.

[74] Xin M., He J., Zhang Y. (2018). Chinese herbal decoction of Wenshen Yangxue formula improved fertility and pregnancy rate in mice through PI3K/Akt signaling. *Journal of Cellular Biochemistry*.

[75] Latifi Z., Nejabati H. R., Abroon S. (2019). Dual role of TGF-*β* in early pregnancy: clues from tumor progression. *Biology of Reproduction*.

[76] Sporn M. B. (1999). TGF-*β*: 20 years and counting. *Microbes and Infection*.

[77] Liu W., Huang Y., Huang G. (2017). Relationship of SOCS3 and TGF-*β* with IDO expression in early pregnancy chorionic villi and decidua. *Experimental and Therapeutic Medicine*.

[78] Holzer U., Rieck M., Buckner J. (2006). Lineage and signal strength determine the inhibitory effect of transforming growth factor *β*1 (TGF-*β*1) on human antigen-specific Th1 and Th2 memory cells. *Journal of Autoimmunity*.

